# Instrumented gait analysis: a measure of gait improvement by a wheeled walker in hospitalized geriatric patients

**DOI:** 10.1186/s12984-017-0228-z

**Published:** 2017-02-27

**Authors:** Samuel Schülein, Jens Barth, Alexander Rampp, Roland Rupprecht, Björn M. Eskofier, Jürgen Winkler, Karl-Günter Gaßmann, Jochen Klucken

**Affiliations:** 1 Geriatrics Centre Erlangen, Waldkrankenhaus St. Marien, Erlangen, Germany; 2 Department of Molecular Neurology, University Hospital Erlangen, Friedrich-Alexander-Universität (FAU) Erlangen-Nürnberg, Schwabachanlage 6, 91054 Erlangen, Germany; 3 Digital Sports Group, Pattern Recognition Lab, Department of Computer Science, FAU, Erlangen, Germany; 4 ASTRUM IT GmbH, Erlangen, Germany; 5 Institute of Psychogerontology, FAU, Erlangen, Germany

**Keywords:** Gait analysis, 4-wheeled walker, Geriatric patients, Risk-of-falling

## Abstract

**Background:**

In an increasing aging society, reduced mobility is one of the most important factors limiting activities of daily living and overall quality of life. The ability to walk independently contributes to the mobility, but is increasingly restricted by numerous diseases that impair gait and balance. The aim of this cross-sectional observation study was to examine whether spatio-temporal gait parameters derived from mobile instrumented gait analysis can be used to measure the gait stabilizing effects of a wheeled walker (WW) and whether these gait parameters may serve as surrogate marker in hospitalized patients with multifactorial gait and balance impairment.

**Methods:**

One hundred six patients (ages 68–95) wearing inertial sensor equipped shoes passed an instrumented walkway with and without gait support from a WW. The walkway assessed the risk of falling associated gait parameters velocity, swing time, stride length, stride time- and double support time variability. Inertial sensor-equipped shoes measured heel strike and toe off angles, and foot clearance.

**Results:**

The use of a WW improved the risk of spatio-temporal parameters velocity, swing time, stride length and the sagittal plane associated parameters heel strike and toe off angles in all patients. First-time users (FTUs) showed similar gait parameter improvement patterns as frequent WW users (FUs). However, FUs with higher levels of gait impairment improved more in velocity, stride length and toe off angle compared to the FTUs.

**Conclusion:**

The impact of a WW can be quantified objectively by instrumented gait assessment. Thus, objective gait parameters may serve as surrogate markers for the use of walking aids in patients with gait and balance impairments.

**Electronic supplementary material:**

The online version of this article (doi:10.1186/s12984-017-0228-z) contains supplementary material, which is available to authorized users.

## Background

In an increasing ageing society reduced mobility is one of the most important factors that limit activities of daily living and overall quality of life [1]. The ability to walk independently is crucial for the individual mobility, but is increasingly restricted by numerous diseases that impair gait and balance in elderly people [2, 3]. Gait and balance disorders are among the most frequent impairments in older adults. They are usually of multifactorial origin, result in the loss of independence, and limited quality of life of patients, and in particular contribute to an increased risk of falling [4–7]. Approximately 30% of adults aged 65 years and older report falls at least once a year [8], with severe complications and consecutive comorbidities. Increased risk of falling has been associated with distinct spatio-temporal gait parameters, such as reduced velocity, swing time, stride length, and increased gait variabilities of stride and double support time in elderly people [8–12]. Therefore, these spatio-temporal gait parameters are potential surrogate markers for gait deficits and risk of falling [13, 14].

Several assistive devices are frequently prescribed in gait and balance disorders in order to support the gait and mobility of affected patients [15–18]. Walking aids such as crutches, canes, or 4-wheeled walkers (WW) are suitable to reduce the dependency on care-givers and also to decrease the burden of care allowing the patient to remain functionally independent and mobile. Thus, assistive devices have become an indispensable and well accepted part within rehabilitation programs since the 1990s [19, 20]. Even though the benefit of walking aids such as WW is generally well accepted [15, 21–23], only little is known about the impact of these devices on gait parameters as objective measures in geriatric patients with gait and balance disorders.

Recent technology developments in instrumented gait analysis provide stance-phase derived spatio-temporal gait parameters derived from well-established instrumented walkways [8, 24], or novel inertial sensor based gait analysis systems that are able to also assess swing phase related gait parameters such as foot angles at the beginning (toe off) or the end (heel strike) of the swing phase, and the toe clearance which might be also important for an increased risk of falls [25–29]. Thus, instrumented gait analyses are easy applicable and enable objective measures of gait impairments and therapeutic effects for example in disease modifying therapies in Parkinson’s disease [30, 31], ataxia [32], multiple sclerosis [33], people with type II diabetes mellitus [34] and effects of lower limp amputations [35].

Two aims were addressed in the present observational study: (I) to investigate whether instrumented gait analysis derived gait parameters are able to objectively measure the effect of using a WW in hospitalized geriatric patients with gait and balance impairment of multifactorial origin, and (II) to identify gait parameters as surrogate markers for the use of a WW. To assess potential confounding familiarization effects we compared first time users (FTUs) with patients who already used a WW (FUs) even before they were admitted to the hospital.

## Methods

### Patient recruitment and characteristics

**Fig. 1:**
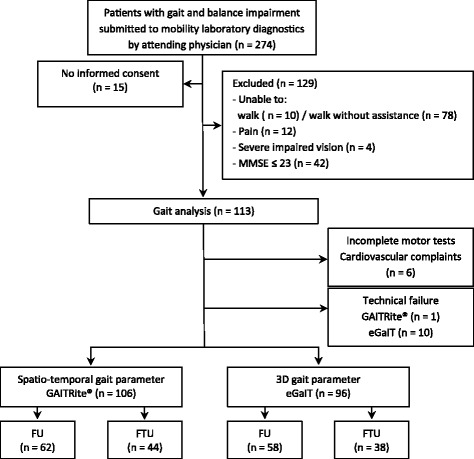
Patients flow chart. MMSE = Mini Mental State Examination; FTU = first time wheeled walker use; FU = frequent wheeled walker user; eGaIT = embedded Gait analysis using intelligent technology

Between April 2012 and May 2013, 274 hospitalized patients routinely submitted to gait and mobility assessment were included in this observational study and subjected to instrumented gait analysis during standardized functional mobility laboratory testing at the Geriatrics Centre of the Waldkrankenhaus St. Marien, Kongregation der St. Franziskusschwestern Vierzehnheiligen, Erlangen, Germany (Fig. 1). Geriatric hospital standard procedures account for multifactorial gait alteration, and subject all patients with signs of gait and balance impairment to laboratory functional mobility assessment. It includes a comprehensive basic geriatric assessment battery [36] and standardized clinical and functional mobility screening as recommended by the Medical Association for the Promotion of Geriatrics in Bavaria, Germany (AFGiB). Besides testing for visual impairment, orthostatic hypotension, and peripheral neuropathy, functional tests in particular include the Timed up and go (TUG), postural stability, and gait analysis. This standard mobility assessment is applied to all hospitalized patients with gait and balance impairment that are frequently use a WW (FUs), but also to patients that use a WW for the first time (FTUs).

In our patient cohort, gait and balance impairment was of multifactorial origin and typically associated with neurodegenerative diseases, peripheral (diabetic) neuropathy, fainting or syncope, vertigo/dizzy spells, attentional or mild cognitive deficits, multimorbidity and polypharmacy (>6 medications per day), and history of falls. Patients meeting these diagnostic screening criteria were submitted by the attending physician to routine diagnostics at the functional mobility laboratory, and thus enrolled in the present observational study. Participants in our study had an average of 11 (range: 2–19) diagnoses (Table 1). The most frequent were arrhythmia (69.9%), arterial hypertension (69.1%), gait impairment/fall proneness (53.6%), coronary artery disease (40.6%), and hypercholesterolemia/dyslipedemia (26.8%). Patients (at least 65 years old) were included if they were able to walk 10 m without assistance, understand and follow verbal instructions. Patients were not included in the study if they exhibited indications of severe pain, impaired vision (<0125/40 Distance 5 m), severe cognitive impairment (Mini-Mental State Examination (MMSE) score ≤ 23) [37], acute injuries, or self-limiting conditions preventing them from performing the tests such as febrile illness, severe headache, fatigue, vomiting and exsiccosis.

**Table 1:** Patient characteristics for total- and subgroups

Variable	Total (*n* = 106)		FU (*n* = 62)		FTU (*n* = 44)		*p*-value
Gender (W/M)	60/46		37/25		23/21		0.448^3^
Age *(years)*	81.7 ± 6.2	(68.5 – 95.4)	81.8 ± 6.4	(68.5 – 95.1)	81.5 ± 5.9	(72.2 – 95.4)	0.815
Height *(cm)*	164.0 ± 9.8	(144 – 184)	162.3 ± 10.0	(144 – 182)	166.4 ± 9.2	(150 – 184)	0.035
Weight *(kg)*	71.4 ± 14.4	(43 – 110)	70.5 ± 14.4	(43 – 110)	72.5 ± 14.5	(50 – 108)	0.489
BMI *(kg/m* ^*2*^ *)*	26.5 ± 4.5	(17 – 38)	26.8 ± 4.8	(17 – 38)	26.1 ± 4.1	(20 – 38)	0.442
Leg length *(cm)*	88.9 ± 5.7	(73.3 – 100.5)	88.5 ± 5.8	(73.3 – 100.5)	89.5 ± 5.6	(78.5 – 98.5)	0.375
Clinical characteristics							
MMSE	27.4 ± 1.8	(24 – 30)	27.3 ± 1.8	(24 – 30)	27.6 ± 1.7	(24 – 30)	0.347
GDS	3.3 ± 2.6	(0 – 12)	3.7 ± 2.8	(0 – 12)	2.8 ± 2.2	(0 – 9)	0.081
FES-I score	31.8 ± 10.7	(16 – 61)	33.6 ± 10.9	(16 – 61)	29.1 ± 9.9	(16 – 56)	0.036
No of medication^1^	8.9 ± 3.0	(2 – 18)	9.4 ± 3.1	(2 – 17)	8.3 ± 2.9	(2 – 18)	0.315^3^
No of diagnosis^2^	11.3 ± 4.0	(2 – 19)	12.1 ± 3.7	(3 – 19)	10.2 ± 4.0	(2 – 19)	0.104^3^
Fall history							
Non fallers	52	49.1%	28	45.2%	24	54.5%	0.246^3^
Fallers	34	32.1%	19	30.6%	15	34.1%	
Recurrent fallers	20	18.9%	15	24.2%	5	11.4%	
Functional characteristics							
POMA	21.1 ± 3.8	(12 – 28)	20.2 ± 3.5	(12 – 27)	22.1 ± 3.9	(12 – 28)	0.009
TUG	20.1 ± 8.1	(9 – 48)	22.5 ± 8.4	(11 – 48)	16.6 ± 6.5	(9 – 40)	<0.001
BARTHEL	53.9 ± 10.8	(30 – 80)	52.5 ± 9.8	(30 – 80)	55.9 ± 11.8	(30 – 80)	0.108

Values are mean ± standard deviation and (range); Fall history = Total number and percentage. *FU* = frequent wheeled walker user, *FTU* = first time wheeled walker user, *BMI =* Body-Mass-Index, *MMSE* = Mini Mental State Examination (range 0 – 30), *GDS* = Geriatric Depression Scale (range 0 – 15); FES-I = Falls Efficacy Scale International (range 16 – 64); Fall history (past 12 months): Non fallers = 0 falls; fallers = 1–2 falls; recurrent fallers = ≥ 3 falls; *POMA ﻿=* Performance Oriented Mobility Assessment (range 0 – 28), *TUG* ﻿= Timed up & Go, *BARTHEL* = Hamburg Classification Manual for the Barthel Index in geriatrics (range 0 – 100). ^1^Number of medications and ^2^diagnosis treated during duration of stay. *P*-value for unpaired *t*-test (FTU; FU); or ^3^Pearsons’s chi-square test for independence

One hundred thirteen patients met the inclusion criteria and 106 completed the instrumented gait tests (Fig. 1). Six patients were excluded during gait analysis because of cardiovascular complaints, orthostatic hypotension, or general discomfort. Due to technical failure, data from one patient examined using the instrumented walkway and from 10 patients using eGaIT had to be excluded.

All patients gave informed consent in agreement with the 1964 declaration of Helsinki. The study was approved by the Clinical Ethics Committee of the Faculty of Medicine, Friedrich-Alexander University Erlangen-Nürnberg (FAU), Germany (Re.No.4208).

### Clinical assessment

Cognition was examined using the Mini-Mental State Examination (MMSE) [38]. Depression was rated using the Geriatric Depression Scale (GDS, total score range 0–15 with scores ≥ 5 indicating possible depression) [39]. Fear of falling was assessed using the Falls Efficacy Scale International questionnaire (FES-I), total score range 16–64, with scores ≥ 28 indicating high fall concerns [40, 41]. Number of falls during the past 12 months was assessed during a semi-structured interview as suggested by Freiberger & Vreede, 2011 [42]. We classified “no fallers”, “fallers” (1–2 falls/year), and “recurrent fallers” (≥3 falls/year). To assess Activity of Daily Living (ADL), performance was assessed using the Hamburg Classification Manual for the Barthel Index (BARTHEL, range 0–100); scores 0–30 indicating dependence on care, 35–80 need for help and 85–100 needs help selectively to independence [43]. Evaluation of functional mobility associated with increased risk of falling was assessed with the 28 point version Performance-Oriented Mobility Assessment (POMA) [44]. The 3 m Timed “Up & Go” test (TUG) was used to assess mobility and functional performance [11, 45, 46].

### Gait analysis

Objective gait analysis of WW assisted gait tasks was performed by two complementary instrumented gait analysis systems: gait parameters were recorded and quantified by I) an instrumented walkway (GAITRite®) [8, 24], and II) an inertial sensor based mobile gait analysis system (eGaIT – embedded gait analysis system using intelligent technology) [26, 31, 47, 48].

### GAITRite walkway derived gait parameter

**Fig. 2:**
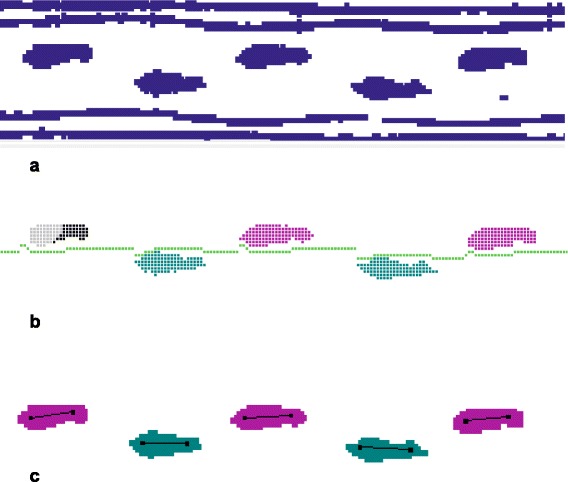
GAITRite® recorded footprint and 4-wheeled walker track signals of a patient (**a**). After manual removal of the WW tracks the use of the automated removal tool of the GAITRite® software caused errors in the calculation of gait parameters du to mislabeling of footprints. Therefore footprint identification was confirmed and adjusted manual by an observer blinded to the data source. The footfalls turn color when the sensors are activated and confirmed by the examiner. Gray = deactivated sensors, black = manually identified sensors (**b**). Green = activated and calculated sensors of the left foot, magenta = activated and calculated sensors of the right foot (**c**)

The five spatio-temporal risk of falling associated gait parameters: (1) *velocity* (cm/s) [9, 12, 49, 50], (2) *swing time (s)* [12], (3) *stride length* (cm) [9, 12], (4) *stride time variability* (% CV) [8, 51, 52], and (5) *double support time variability* (% CV) [9, 12] were recorded by an instrumented walkway (GAITRite® walkway; Model Platinum CIR Systems Inc., 8 John Walsh Blvd., Peekskill NY 10566 WA) according to the guidelines of the European GAITRite Network Group [53]. The use of the WW interfered with the correct automated detection of gait parameters because the pressure imprint of the WW was falsely identified as a footprint by the GAITRite® system (Fig. 2). Therefore footprints of the gait task with WW were manually identified by a researcher blinded to the source of data and gait parameters were recalculated [26].

### eGaIT derived gait parameter

**Fig. 3:**
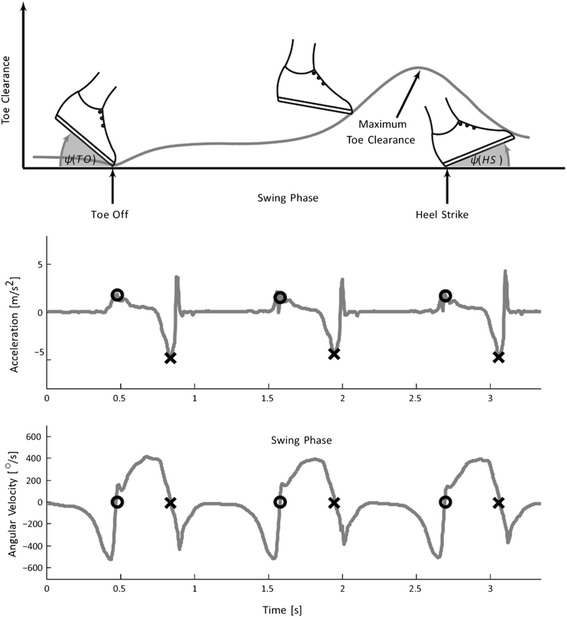
Inertial sensor based gait parameter extraction paradigm. The upper plot shows an example signal of angles at toe off and heel strike and toe clearance during the swing phase. The middle plot show an example signal of acceleration in up and down direction and lower plot shows angular velocity in sagittal plane. The gait events heel strike (HS, X) and toe off (TO, O) are marked in the plots. Heel strike is determined from the negative peak in the acceleration signal and toe of from the zero crossing in the angular velocity signal

In addition to the spatio-temporal gait parameters we were able to measure complementary swing phase associated gait parameters from the sagittal plane including (1) *toe off angle*: defined as the foot angle in sagittal plane at the toe off event during gait cycle; (2) *heel strike angle*: defined as the foot angle in sagittal plane at the temporal event when the heel hits the ground; and (3) *maximal toe clearance*: defined as the maximum distance between toe and ground during swing phase (Fig. 3) using an instrumented mobile gait analysis system (eGaIT) [26]. The mobile eGaIT gait analysis system consists of inertial motion sensors (3-axis gyroscope (range ± 500°/s) and a 3-axis accelerometer (range ± 6 g; Shimmer 2R, Shimmer Sensing, Dublin, Ireland) laterally mounted to the heel of both shoes (Fig. 3). In contrast to the GaitRite system, the inertial motion sensors provide accelerometer and gyroscope data of the swing phase, thus allowing to compute the three additional gait parameters as previously described [26]. Briefly, by using stride segmentation [47] and gait parameter calculation algorithms [25, 54, 55], *toe off* and *heel strike angles* were automatically calculated from the raw sensor data in degree (°), and *maximal toe clearance* in cm (Fig. 3). Calculation of these parameters has been previously validated using an optical motion capture system (Vicon Motion Systems Ltd., UK) [48]. Using the eGaIT system foot angle in sagittal plane is calculated with a mean absolute error of 2.49° ± 1.21° and toe clearance with a mean absolute error of 1.69 cm ± 1.21 cm as described [48].

### Gait test paradigm

All patients underwent standardized gait assessment over a straight 10 m walking distance crossing the GAITRite walkway wearing the inertial motion sensor equipped shoes. The complete gait assessment included two gait tests: (1) *Normal walking:* Patients were instructed to cross the instrumented walkway unsupported at their self-selected “normal” walking speed (2) *walking with a WW:* The walkway was crossed with the support of the WW (Rollator B, Modell 4003, Bischoff & Bischoff GmbH, Karlsbad, Germany) at “normal” speed as described in (1). All patients used the same WW model and correct handle height was adjusted individually by measuring the distance from the floor to the joint line of the wrist. Thereby the patient stood upright with arms relaxed at the side of the body [16, 56]. After the WW had been adjusted to the patient, FTUs familiarized themselves with the WW walking for approximately 5 min. No specifically instructed practice trials were performed. Both FTU´s and FUs were given the instructions to walk with an upright posture while maintaining the hip between both handles of the WW. Patients were allowed to rest between the tasks if needed. Testing was immediately stopped if a subject showed signs of discomfort. Patients initiated and terminated each gait task 2 m before and after the walkway to enable steady-state walking over the GAITRite walkway.

### Statistics

Comparative statistics on patient characteristics were calculated using Pearson’s Chi-Square or Student’s *T*-test as indicated. The effect of WW usage (main within-subject effect) was calculated by ANOVA with repeated measures using FU vs. FTU as interaction effect. Statistical tests were carried out using the IBM SPSS statistics 23 program for Microsoft Windows. The level of significance was set at *p* ≤ 0.05.

## Results

The mean age of the cohort (*n* = 106) was 81.7 ± 6.2 years with a balanced gender (56.6% women). 41.5% of all participants were using the WW for the first time (FTUs). No differences in gender, age, height, and weight or body mass index were noted across the two groups (Table 1). The number of diagnoses and medication was slightly higher in the FUs without reaching significance. Psychometric results showed that cognitive function did not differ between FUs and FTUs (*p* = 0.347, MMSE score). No or only mild levels of depression was observed in all patients. 16 patients in the FU group were mildly depressed (GDS > 5), and only 7 in the FTU subgroup without reaching statistical significance (*p* = 0.207, Pearson’s Chi-square test).

Functional mobility assessment battery revealed stronger signs of impairment in patients that were used to a WW (FUs) compared the FTUs in several parameters. Fear of falling assessed by the FES-I questionnaire revealed an overall increased fear of falling level (mean: 31.8 ± 10.7 SD, cut off ≥ 28), which was even more pronounced in the FUs compared to the FTUs. Numbers of falls in the past 12 months were recorded and classified into three groups. In total, 49.1% reported no falls, 32.7% experienced 1–2 falls and 18.9% reporting three or more falls. The percentage of frequent fallers was higher in the FU subgroup without reaching statistical significance. Functional impairment was higher in the FUs compared to the FTU as reflected by the POMA (*p* = 0.009) and the TUG (*p* < 0.001) scores. Also, the Barthel index indicating functional dependency was lower in the FUs compared to the FTUs without reaching significance (*p* = 0.108). In summary, FU patients accustomed to a WW showed higher functional impairment than FTU patients not using a WW.

The complete cohort of patients with different levels of gait impairment showed decreased spatio-temporal gait parameters recorded from the 10 m walk (*normal walk*) compared to historical normative values derived from a healthy aged cohort reported by Hollman et al.: *velocity (109.5 cm/s)*, *swing time (0.40 s)* and *stride length (123 cm)* [57]. Also, several spatio-temporal gait parameter impairments correlated to the functional scores FES-I, Tinetti/POMA and TUG, but not with the Barthel index or the psychometric scores MMSE or GDI (Additional file 1: Table S1) indicating the association of the functional immobility levels within the patient cohort to the gait parameter impairments which was also in line with the routine admission procedure to functional mobility tests within the geriatric hospital. Within the present cohort, velocity and stride length were significantly lower in the FUs compared to FTUs (Table 2). Also, the novel objective inertial sensor-based mobile sagittal plane gait parameters (e.g. *toe off* and *heel strike angles,* and *maximal toe clearance)* were significantly lower in FUs (Table 2).

**Table 2:** Gait parameters in FU and FTU

		Normal Walk						
		Total		FU		FTU		Significance
Variable		*n* = 106		*n* = 62		*n* = 44		*p*-value
Spatio-temporal gait parameters		Mean	± SD	Mean	± SD	Mean	± SD	*p*
Velocity	(cm/s)	73.1	±21.3	65.8	±20.5	83.4	±18.0	<0.001
Swing time	(s)	.38	± .06	.37	± .06	.39	± .05	0.057
Stride length	cm	89.2	±21.8	81.6	±22.6	100.0	±16.3	<0.001
Stride time variability	(%CV)	4.2	±2.5	4.7	±2.8	3.4	±1.7	0.005
Double support time variability	(%CV)	7.6	±4.0	8.0	±4.6	7.0	±3.0	0.234
sagittal plane gait parameters		*n* = 96		*n* = 58		*n* = 38		
Toe off angle	(°)	42.1	±8.1	40.6	±8.8	44.8	±6.2	0.005
Heel strike angle	(°)	11.8	±4.9	10.7	±4.9	13.5	±4.5	0.009
Max. toe clearance	(cm)	6.1	±2.0	5.6	±1.9	6.9	±2.1	0.003

*FTU* = first time WW user, *FU* = frequent WW user, (°) = degree, (*cm*) = centimeter, (*% CV*) = coefficient of variation, calculated by the formula: [CV (%) = SD/Mx100]; Significance was calculated for the difference in gait parameters between FU and FTUs (unpaired *t*-test, 2-sided)

We next asked whether the use of a WW improved risk of falling associated spatio-temporal and sagittal plane gait parameters. Thus, all patients performed a *normal walking* without a WW (WW -) and a second walk using a *4-wheeled walker* (WW +). Both *velocity* and *stride length* increased by 1.18 fold (main effect - Table 3). *Swing time* increased by 1.07 fold. *Stride time and double support time variabilities,* however, were not influenced by WW use (Table 3). The use of WW also improved the sagittal plane gait parameters *toe off* and *heal strike angles*. No change in *maximal toe clearance* was observed in all patients.

**Table 3:** Gait parameter improvement by WW in FU and FTU

		Fold improvement by WW				
Variable		Total	FU	FTU	Significance	
					Main effect	Interaction effect
		Mean ± SD	Mean ± SD	Mean ± SD	F (*p*)	
Spatio-temporal gait parameters		*n = 106*	*n = 62*	*n = 44*		
Velocity (cm/s)	x	1.18 ± 0.29	1.25 ± 0.34	1.06 ± 0.16	44.51 (<0.001)	10.91 (0.001)
Swing time (s)	x	1.07 ± 0.15	1.09 ± 0.17	1.04 ± 0.10	20.61 (<0.001)	1.87 (0.174)
Stride length (cm)	x	1.18 ± 0.28	1.25 ± 0.32	1.08 ± 0.15	56.13 (<0.001)	8.96 (0.003)
Stride time variability (% CV)	x	1.04 ± 1.80	1.10 ± 2.28	0.95 ± 0.74	12.68 (0.001)	8.63 (0.004)
Double support time variability (% CV)	x	1.04 ± 1.24	1.14 ± 1.52	0.90 ± 0.68	0.31 (0.576	0.54 (0.464)
Sagittal plane gait parameters		*n = 96*	*n = 58*	*n = 38*		
Toe off angle (°)	x	1.09 ± 0.14	1.12 ± 0.16	1.04 ± 0.10	32.88 (<0.001)	6.46 (0.013)
Heel strike angle (°)	x	1.53 ± 2.35	1.57 ± 2.8	1.45 ± 1.45	42.11 (<0.001)	0.37 (0.544)
Max. toe clearance (cm)	x	1.0 ± 0.24	1.05 ± 0.26	0.92 ± 0.18	3.16 (0.079)	6.67 (0.011)

Mean individual fold of change (“x”) by usage of a WW over walk unaided; *FTU* = first time WW user, *FU* = frequent WW user; (°) = degree, *cm* = centimeter, (*% CV*) = coefficient of variation, calculated by the formula: [CV (%) = SD/Mx100]. F-values (1, 104) and *p*-values are presented for the main effect = walk unaided v.s. WW walk; Interaction effect = walk unaided v.s. WW walk * FU v.s FTU

Since gait parameters improved by using a WW in all patients, we wanted to know whether patients that were not used to a WW before (FTUs) showed a similar improvement of gait parameters compared to FUs to evaluate potential confounding effects of low familiarization. In addition, we addressed the question, of whether geriatric patients with gait and balance impairment that were less functionally impaired (FTUs) exhibit distinct gait parameter changes in response to a WW use compared to FUs (Table 3 – interaction effect). Importantly, the tendency of improvement was similar between FTUs and FUs for all spatio-temporal gait parameters and for the foot angles. Nevertheless, the improvement in velocity, stride length, and toe off angle was significantly higher in FUs compared to FTUs (Table 3 – interaction effect). Swing time improvement was similar in both groups, whereas *stride time variability* and *double support time variability* were not changed in both groups (Table 3). Surprisingly, the change of *maximal toe clearance* was significantly different between FUs and FTUs (Table 3, interaction effect). By evaluating the effect of a WW in both groups independently revealed even a significant decrease of *maximal toe clearance* in FTUs by 8% (*p* =0.005, unpaired *T*-test), while the FUs showed no difference.

## Discussion

In this cross-sectional cohort study of hospitalized geriatric patients with multifactorial gait impairment, we investigated the impact of a WW on risk-of-falling associated and additional sagittal plane gait parameters. We demonstrated that the use of a WW improved distinct gait parameters in geriatric patients. This improvement was stronger in patients that were accustomed to the use of a WW (FU subgroup) compared to first time users (FTU subgroup), also reflecting the higher level of functional gait impairment in FUs. Importantly, less affected FTUs who were not familiarized with the use of a WW showed similar patterns of gait parameter improvement as FUs, although to a milder extent. In addition, the ability to assess sagittal plane gait parameters by inertial sensor-based gait analysis (eGaIT) allowed us to identify novel objective gait parameters that might be associated to risk of falling and also improved by the use of a WW in geriatric patients.

Gait impairment in older adults substantially reduces the quality of life, limits mobility, and is associated with increased risk of falling [2]. Multifactorial gait impairment in geriatric patients eventually leads to slower and more instable gait reflected by reduced gait speed and increased stride-to-stride variability of gait parameters [3]. At present, standardized clinical assessment procedures of hospitalized geriatric patients include a broad set of screening tools [36] not only focusing on an individual diagnosis leading to gait impairment, but rather addressing functional impairments and reduced quality of life [13]. Instrumented functional assessment of gait is able to complement diagnostic screens by providing objective gait parameters reflecting overall gait impairment independent of the diagnosis but in correlation with the functional assessment scores (Additional file 1: Table S1), thus, being relevant for therapeutic interventions and monitoring [7]. In the present study, geriatric patients with different signs of gait impairment were subjected to instrumented gait tests using an instrumented walkway and inertial sensor-based gait analysis. Here, we showed that all patients have dysfunctional gait characteristics that could be quantified by objective gait parameter assessment. In particular, velocity was reduced compared to historical normative data (82.9 cm/s vs. 109.5 cm/s) [57] in all patients, independent of the distinct diagnoses. The multimorbid and frail characteristics of our patient cohort are indicated by a mean number of 11 diagnoses and the patient’s higher functional mobility impairment, as assessed by POMA and TUG (Table 1) and also a stronger level of gait parameter impairment (Table 2). A decline in velocity often reflects a reduction in physical mobility in elderly people. Gait velocity values for healthy older adults have been reported around 1.3 m/s [49]. Healthier aging and survival in community-dwelling elderly has been associated with velocities faster than 0.8 – 1.0 m/s, and the likelihood of frailty and poor overall health strongly correlates with gait velocity slower than 0.6 m/s [58–60]. In our cohort of gait impaired patients, gait speed was reduced in both subgroups. Patients that were accustomed to the use of a WW showed even stronger reduced velocity (Table 2). This may be explained by the presence of more severe gait impairment in patients depending on the use of a WW for a longer period, which is also consistent with previous findings reporting that community-dwelling elderly using walking aids such as a cane, crutch, or WW had a significant slower gait during unassisted walking [14].

The present study focused in particular on established risk of falling associated gait parameters velocity, swing time, stride length, as well as stride time and double support time variabilities. In addition, we were also able to record additional sagittal plane gait parameters that might also be associated to falling [26]. Stride length and velocity increased by the use of a WW in the present cohort. It has been shown that enlarged stride length leads to an increase in velocity [14, 15].

Interestingly, we could not detect difference in the two gait parameters stride time and double support time variabilities. Gait variabilities have been associated not only with irregular and unstable gait but also with an increased risk of falling [9, 12, 61], frailty [51], and with other neurodegenerative diseases [62, 63]. It is important to note that gait variabilities in the present laboratory gait test are deducted from only 10 m walking distance. This clearly limits the informative value of gait variabilities as a surrogate marker which is also reflected by the large deviations observed in the present cohort (Table 2 and 3). Ideally, longer gait sequences would be required to assess more reliable gait variabilities, but might also lead to exhaustion or fatigue, in particular in a cohort with already severe gait and mobility impairments. Thus, based on this limitation of the study our data support the hypothesis that gait variabilities cannot be measured form only 11–13 strides as evaluated in the present gait analysis setting. Future analysis concepts using numerous gait sequences from continuous gait monitoring over days and weeks might help to solve this principle challenge. However, our findings indicate that a 10 m walk as a standardized test frequently used in routine clinical diagnostics has already the potential to improve the diagnostics for WW usage in clinical care.

Interestingly, not only the spatio-temporal falling associated gait parameters improved by the use of a WW in all patients, but also the swing phase associated gait parameters in the sagittal plane such as toe off and heel strike angle. Increasing hip flexion and extensor moments, the reduction in knee flexion in early stance, and less ankle range of motion (ROM) and ankle plantar flexor power were reported as age related adaption of gait [64–68]. Increasing stride length and velocity in FUs and FTUs using the WW may result in an increase in ankle ROM and thus affect toe off and heel strike angles. The increase in heel strike angle during WW use could be explained by an involuntary reactive deceleration through an accentuated use of the heel protecting the user against unwanted further acceleration.

Unexpectedly, investigating swing phase associated gait parameters in FTUs showed a reduction in maximal toe clearance. It has been described that patients with impaired gait characteristics exhibit a punting or tamping behavior in order to actively increase the maximal toe clearance [69]. Under healthy conditions, a person walking with an upright posture relies only on his feet for a base of support (BOS). In this position, the somatosensory input is received primarily from the lower extremities. During normal gait performance without a WW, the reciprocal swing of the upper extremities could allow them to constantly be alert and ready to help maintain the center of mass (COM) over the BOS when balance is affected. However, using a WW might change these body mechanics. The alternating arm-swing will be lost by WW usage and bilateral somatosensory input from the lower extremities may gradually adapt to quadrilateral somatosensory inputs from both lower and upper extremities. Furthermore, the use of an WW allows the upper extremities to compensate for weak lower extremity muscles and allows total bodyweight to be supported by upper and lower extremities rather than the lower extremities alone [70]. Thus, using a WW leads to a considerable enlargement of the base of support (BOS) between the patient’s feet and the four wheels of the device. This allows to position the body’s COM over the BOS to achieve static postural equilibrium [15, 71]. Our findings show that using a WW in FTUs resulted in a reduction in maximal toe clearance. One can speculate that this could be a consequence of the biomechanical and psychological support by the WW where patients experience an unprecedented sense of comfort and safety. It is also not clear, whether and how this also contributes to an increased risk of falling after WW use which has been shown by other authors [72–74].

The WW test paradigm for FTUs in the present study mimics the typical diagnostic test setting, where a patient with gait impairment is routinely subjected to functional mobility testing including the use of a WW. Future studies have to confirm the findings in this selected population where the recruitment of patients with heterogeneity of diagnoses depends on the experience of the attending physician in a more active community-dwelling population. Nevertheless, using this paradigm of patient selection and the complementary approach using mobile and stationary assessed gait parameters we were able to isolate distinct gait characteristics that are especially sensitive for the use of a WW. Therefore, the change of these gait parameters could be used as surrogate markers for the effect of a WW.

The interpretation of our findings that the use of a WW can be objectively measured by instrumented gait parameters is limited by several considerations: For the FTU group familiarization aspects might be confounding factors for the change of gait parameters. It has been reported that first time WW usage can lead to dissatisfaction and requires to get used to the WW [75]. Furthermore, the use of a WW can be stigmatizing and thereby negatively affect the use of a WW. In addition, the importance of an introduction, training in basic functions and handling of the WW prior to first time use has an impact on the applicability of a WW [74, 75]. Even though we included a short time of familiarization with the WW for FTU, we cannot exclude that low familiarization affects the outcome gait parameters. Therefore, it is important that FTUs improved in velocity, swing time, stride length, heel strike- and toe off angle in a similar manner compared to FUs, suggesting that the low level of familiarization did not substantially affect the applicability of gait parameters as surrogate markers for WW use also in the FTU group. Finally, the changes in gait parameter do not provide information on the long term consequences in particular in the light of poorer functional status in the FU compared to the FTUs. Future prospective studies to examine familiarization affects and long term consequences are still needed.

## Conclusion

Using a WW improved distinct risk of falling associated gait parameters in the present hospitalized geriatric patient cohorts. Although gait performance of FTUs and FUs did not reach the level of a normal, healthy and stable gait, our findings indicate the potential of objective gait parameter assessment to improve and complement the diagnostic workup also providing target parameters to monitor intervention efficacy. Future longitudinal studies are warranted to validate the predictive value on known risk of falling associated and novel sagittal plane gait parameters under WW usage.

## Additional file

Additional file 1: Table S1. Correlations analysis between gait parameter and clinical/functional assessments. (DOCX 19 kb)

## Abbreviations

ADL: Activity of daily living
eGaIT: Embedded gait analysis system using intelligent technology
FTU: First time users
FU: Frequent users
GDS: Geriatric depression scale
MMSE: Mini-mental state examination
POMA: Performance-oriented mobility assessment
TUG: Timed up and go
WW: Wheeled Walker
