# Supplementary material for: Instrumented gait analysis: a measure of gait improvement by a wheeled walker in hospitalized geriatric patients
Source: J Neuroeng Rehabil. 2017 Feb 27;14:18. doi: 10.1186/s12984-017-0228-z (PMC5327552; doi:10.1186/s12984-017-0228-z)
Supplement: Additional file 1: Table S1. — Correlations analysis between gait parameter and clinical/functional assessments. (DOCX 19 kb) [file 12984_2017_228_MOESM1_ESM.docx]

Supplement Table: Correlations analysis between gait parameter and clinical/functional assessments

|  | FES-I | | Tinetti/POMA | | TUG | | BARTHEL | | MMSE | | GDS | |
| --- | --- | --- | --- | --- | --- | --- | --- | --- | --- | --- | --- | --- |
|  |  | |  | |  | |  | |  | |  | |
|  | **r** | **p** | **r** | **p** | **r** | **p** | **r** | **p** | **r** | **p** | **r** | **p** |
| Velocity | -.36 | .001 | -.40 | .001 | -.54 | .001 | .16 | .098 | .12 | .212 | .08 | .448 |
| Swing time | -.19 | .057 | .24 | .016 | -.25 | .012 | .09 | .385 | .12 | .214 | -.12 | .269 |
| Stride length | -.36 | .001 | .43 | .001 | -.56 | .001 | .16 | .100 | .14 | .167 | -.00 | .981 |
| Stride time variability | .23 | .020 | -.29 | .003 | .31 | .001 | -.02 | .853 | -.03 | .749 | -.03 | .727 |
| Double support time variability | .32 | .001 | -.29 | .003 | .49 | .001 | -.12 | .219 | -.08 | .418 | -.19 | .050 |
|  |  |  |  |  |  |  |  |  |  |  |  |  |
| Toe off angle | .28 | .400 | -29 | .035 | .40 | .003 | -.14 | .318 | .01 | .917 | .14 | .306 |
| Heel strike angle | .02 | .911 | -.07 | .639 | .04 | .780 | .20 | .162 | .16 | .25 | .09 | .520 |
| Max. toe clearance | -.31 | .035 | .345 | .020 | -.41 | .005 | -.02 | .923 | .01 | .963 | .02 | .896 |
|  |  |  |  |  |  |  |  |  |  |  |  |  |
|  |  |  |  |  |  |  |  |  |  |  |  |  |
| The Pearson correlation is significant at the 0.05 level (2 side test)  FES-I = Falls Efficacy Scale International  Tinetti/POMA= Performance-Oriented Mobility Assessment  TUG = Timed Up & Go  BARTHEL = Barthel Index , Hamburg Classification Manual for the Barthel Index in geriatrics  MMSE = Mini Mental State Examination  GDS = Geriatric Depression Scale | | | | | | | | | | | | |
